# Correlation between the EGF gene intronic polymorphism, rs2298979, and colorectal cancer

**DOI:** 10.3892/ol.2013.1481

**Published:** 2013-07-22

**Authors:** VAHID CHALESHI, MAHDI MONTAZER HAGHIGHI, SANAZ SAVABKAR, NEDA ZALI, MOHSEN VAHEDI, MAHSA KHANYAGHMA, GHOLAM REZA JAVADI, HAMID ASADZADE, MOHAMMAD REZA ZALI

**Affiliations:** 1 Basic and Molecular Epidemiology of Gastrointestinal Disorders Research Center, Shahid Beheshti University of Medical Sciences, Tehran, Iran; 2 Gastroenterology and Liver Diseases Research Center, Shahid Beheshti University of Medical Sciences, Tehran, Iran; 3 Department of Epidemiology and Biostatistics, School of Public Health, Tehran University of Medical Sciences, Tehran, Iran; 4 Department of Biology, Science and Research Branch, Islamic Azad University, Tehran, Iran

**Keywords:** colorectal cancer, epidermal growth factor, rs2298979, single nucleotide polymorphism

## Abstract

Colorectal cancer (CRC) is an important disorder that results from genetic and epigenetic alterations in one colonic epithelial cell. Epidermal growth factor (EGF) is critical in the development of tumors in epithelial tissues. Variations in the DNA sequence of the *EGF* gene may be particularly significant with regard to susceptibility to CRC. The present study aimed to investigate the effect of the *EGF* gene single nucleotide polymorphism (SNP), rs2298979, on CRC. In this prospective study, 220 samples were collected from patients with CRC and compared with 220 matched healthy controls. Genotyping was performed using the polymerase chain reaction-restriction fragment length polymorphism (PCR-RFLP) method, and the result was validated by direct sequencing. A significant correlation was observed between the rs2298979 variant in the *EGF* gene and CRC. The frequency of the A/G genotype in the control group was higher than in the patients with sporadic CRC [odds ratio (OR), 0.488; 95% confidence interval (CI), 0.307–0.774; P=0.002]. In this study there were no individuals with a G/G genotype. Although the frequency of the G and A alleles was similar in the healthy control and CRC patient groups, individuals with the A/G genotype were less susceptible to CRC compared with those with the A/A genotype.

## Introduction

Colorectal cancer (CRC) originates from one colonic epithelial cell due to an accumulation of genetic and epigenetic changes that lead to malignancy (1). The incidence of CRC has a varied distribution in the different geographical regions and a significant difference has been observed between developed and developing countries (2,3). A number of studies have shown show that CRC is becoming increasingly common in Asian countries (4,5,6). According to the statistics issued by the Iranian Ministry of Health, ~1,130 patients suffering from CRC succumbed in 2006, and there was no significant difference between the mortality rates of male and female patients (7). The causes of CRC may include genetic and environmental factors. Based on the observed molecular genetic alterations, it has been indicated that CRC is a heterogeneous disease (8). For the development of more appropriate diagnostic methods, there is a requirement for studies to identify new molecular markers in the affected populations. Epidermal growth factor (EGF) is one of the most important factors leading to the development of tumors and plays a significant role in the uncontrolled reproduction of cancerous cells (9). EGF is an important regulator of cell survival, and in numerous types of cancer, including breast, prostate, pancreas, colorectal, lung and head and neck, an increase in *EGF* and ErbB, a member of the EGF receptor family, has been demonstrated (10–12). The *EGF* gene is located on chromosome 4q25–27 and produces various transcripts, the largest of which has 24 exons and 23 introns (12,13). Genetic factors play a key role in the susceptibility to diseases, resistance against medications and interference in the interaction with peripheral factors. Single nucleotide polymorphisms (SNPs) are the most common genetic changes (14). As the polymorphisms of the *EGF* gene affect the susceptibility to numerous types of cancer, the majority of the case-control studies on *EGF* genes have been conducted on exonic polymorphisms and untranslated regions (UTRs) (12,15–17). In the present study, the hypothesis that the noncoding polymorphism, rs2298979, may be used to predict the susceptibility to CRC was tested in the Iranian population.

## Materials and methods

### Study population

Genetic analysis was conducted on a population of 220 patients suffering from sporadic CRC and 220 normal individuals who had been referred to the Research Institute for Gastroenterology and Liver Diseases (RIGLD), Taleghani Hospital, Shahid Beheshti University of Medical Sciences (Tehran, Iran). Patients with a family history of hereditary non-polyposis CRC (HNPCC) and familial adenomatous polyposis (FAP) were excluded from this study. The patients and healthy individuals were all of Iranian nationality. A colonoscopy was performed on all participants in the patient and control groups, the medical normality of the control group was confirmed and the histological diagnosis of the pathologist was approved by the gastroenterologist. The parameters of age, gender and cigarette smoking status were controlled in the patient and control groups and the stage of CRC was determined in the patient group. The tumor stage was classified according to the tumor-node-metastasis (TNM) classification of the Union for International Cancer Control (UICC; Table I). This study was conducted under the approval of the ethics committee of the Gastroenterology and Liver Diseases Research Center, Shahid Beheshti University of Medical Sciences (Tehran, Iran).

### DNA extraction

Subsequent to obtaining a letter of consent from each individual, 5 ml peripheral blood was collected and stored at 4ºC in a bottle containing EDTA. Genomic DNA was extracted as soon as possible following sampling using the standard salting out method (18). The quality of the extracted DNA was then assessed using a NanoDrop spectrophotometer (NanoDrop Technologies, Inc., Wilmington, DE, USA).

### EGF rs2298979 gene polymorphism genotyping

Two specific primers were designed. The polymorphism was determined using the polymerase chain reaction-restriction fragment length polymorphism (PCR-RFLP) method. The characteristics and sequence of the primers are shown in Table II. The 768-bp DNA fragment that was located in the intron region of the EGF gene was amplified using the specific primers (Table III). The PCR products were digested by *Pci*I endonuclease enzyme (recognition sequence A/CATGT) for 6 h at 37ºC. In order to observe the digested fragments, the RFLP solution was separated on a 3% agarose gel and stained with ethidium bromide.

### Sequencing

**Figure 1:**
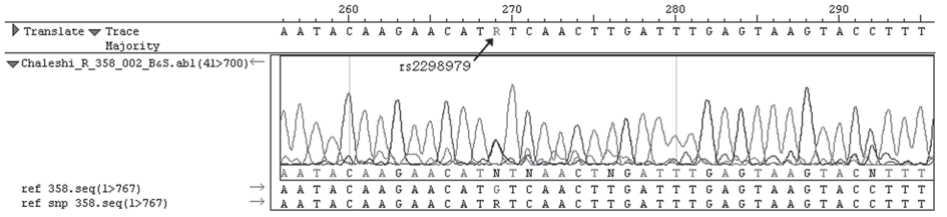
Direct DNA sequencing results for the epidermal growth factor (*EGF*) rs4444903 A/G genotypes.

To confirm the RFLP procedure, 10% of the PCR products were sequenced using the ABI PRISM 3130xL Genetic Analyzer (Applied Biosystems^®^, Invitrogen Life Technologies, Carlsbad, CA, USA) and the chain termination method (Fig. 1).

### Statistical analysis

A Pearson χ^2^ test and Student's t-test were used to calculate the P-value, with P<0.05 considered to indicate a statistically significant difference. The data were analyzed using SPSS statistical software version 13 (SPSS, Inc., Chicago, IL, USA).

## Results

Following enzymatic digestion, it was revealed that the size of the PCR products for the A/A genotype fragment was 768 bp in length, while the three fragments for the A/G genotype were 768, 508 and 260 bp, respectively. The genotype frequency percentages of the rs2298970 polymorphism for the patients with CRC were 28.6% for the A/A homozygote genotype and 71.4% for the A/G genotype. Moreover, the genotype frequency percentages of the controls were 16.4% for the A/A genotype and 83.6% for the A/G genotype. In the entire patient and control group population, the G/G genotype was not observed. Further details and frequency percentages of the A and G alleles for the patient and control groups are shown in Table IV. According to the results of the present study, there was a significant correlation between the A/G genotype of the rs2298979 polymorphism and a decreased risk of CRC compared with the control group [odds ratio (OR), 0.488; 95% confidence interval (CI), 0.307–0.774; P=0.002]. However, no significant correlation was observed among the genotypes of the patients in terms of the stage parameters (Table V).

## Discussion

*EGF* has a significant role in cell proliferation, differentiation and tumorigenesis of epithelial tissues (19). The action of a cell continuing along its intended survival or death pathway is affected by changes in the environmental signals. One of the most important signals from the periphery of the cell inducing cell survival is that of EGF*,* which attaches to the cell receptor (ErbB receptor family) and stimulates intercellular pathways. EGF signaling pathways are regulated by the concentration of EGF present (11). It has been demonstrated that the level of EGF in the plasma is significantly correlated with CRC (20). Polymorphisms of the *EGF* gene have also been shown to be correlated with several other types of cancer. Early studies were conducted to investigate the +61A/G polymorphism in the 5′UTR of the *EGF* gene. The results revealed that the polymorphism was correlated with cancer in individuals carrying the G allele of the +61A/G polymorphism of the *EGF* gene (12,15,16). To the best of our knowledge the correlation between the rs2298979 polymorphism of the *EGF* gene and sporadic CRC has been studied for the first time in the present study. The rs2298979 polymorphism is located in intron 1 of the *EGF* gene. In the present study, it was observed that the carriers of the A/G genotype in the patient group were less susceptible to CRC in comparison with the carriers of the hereditary A/A genotype. The mechanism of this correlation is unknown. The region where the SNP is located may be part of the regulatory sequence (21). The majority of studies on human genome sequences focus on protein coding regions (exons) (22). The protein coding regions in combination with the UTRs account for only 2% of the human genome. It has been revealed that the non-coding regions of the genome are effective in development, natural physiology and pathogenic processes (22). In addition, it has been demonstrated that there are SNPs present in intron regions that have a significant correlation with cancer and different types of diseases. In a study by Millar *et al*(23), it was revealed that the +1169A/T polymorphism located within intron 4 of the growth hormone I (GHI) gene was significantly correlated with the decrease in the circulation of growth hormone and the risk of cancer of the large intestine (23). Furthermore, Li *et al*(24) investigated the affect of two polymorphisms, +45G15G (T/G) and +276 (G/T), located in exon 1 and intron 4 of the adiponectin gene, respectively, in patients suffering from polycystic ovary syndrome (PCOS). No significant correlation was revealed between the disease and the +45G15G (T/G) polymorphism. By contrast, there was a statistically significant correlation between the +276 (G/T) polymorphism, located in intron 4, and PCOS disease (P=0.0126) (24). In conclusion, the present study demonstrates that the carriers of the A/G genotype among patients with CRC are less susceptible to the risk of cancer in comparison with the carriers of A/A genotype. Further studies on the intron regions of the *EGF* gene, particularly the rs2298979 polymorphism, are required in other populations.

## Figures and Tables

**Table I:** Demographic characteristics of the study population.

Variable	Patients with CRC	Controls
Age, years ± mean	43.12±15.366	59.17±13.615
Gender, n (%)		
Male	124 (56.4)	97 (44.1)
Female	96 (43.6)	123 (55.9)
Smoking, n (%)		
Yes	15 (6.8)	16 (7.3)
No	205 (93.2)	204 (92.7)
Clinical stage, n (%)		
Stage I and II	101 (45.9)	-
Stage III and IV	119 (54.1)	-

Patients with colorectal cancer (CRC), n=220; controls, n=220.

**Table II:** Primer sequence and resulting fragment length for the rs2298979 PCR.

Primer no.	Direction	Primer sequence	% GC	Result (bp)
1	Forward	5′-CATACAATAAACACTCGATAAGCC-3′	37.5	
2	Reverse	5′-ACCTCCAACCAACCATACTACC-3′	50	768

PCR, polymerase chain reaction.

**Table III:** Technical data for the rs2298979 polymorphism detection method.

Factor	Value
PCR reaction condition, no. of cycles	
94ºC for 5 min	1
94ºC for 45 sec	30
65ºC for 40 sec	-
72ºC for 45 sec	-
72ºC for 5 min	1
Master mix, μl	
10× PCR buffer	2.50
MgCl_2_ (50 mM)	0.75
Primer-forward (10 mM)	1.00
Primer-reverse (10 mM)	1.00
dNTP (10 mM)	0.50
Taq polymerase (5 U/μl)	0.50
Water	17.75
Restriction enzyme	*Pci*I
Restriction enzyme time, h	6
Restriction pattern length, bp	
A	768
G	508+260
Agarose gel concentration, %	3

PCR, polymerase chain reaction; dNTP, deoxyribonucleotide triphosphate.

**Table IV:** Colorectal cancer risks correlated with rs2298979 SNP that were examined in the present study.

				OR (95% CI)		
					
Genetics	Patients, n (%)	Controls, n (%)	P-value	Unadjusted	Adjusted	P-value
Genotype						
A/A	63 (28.6)	36 (16.4)	-	1.00 (Reference)	1.00 (Reference)	-
A/G	157 (71.4)	184 (83.6)	0.002	0.488 (0.307–0.774)	0.449 (0.263–0.767)	0.003
Alleles						
A	283 (64.3)	256 (58.2)	-	1.00 (Reference)	-	-
G	157 (35.7)	184 (41.8)	0.062	0.772 (0.588–1.013)	-	-

Patients, n=220; controls, n=220. SNP, single nucleotide polymorphism; CI, confidence interval; OR, odds ratio.

**Table V:** Tumor-stage specific distribution of EGF rs2298979 genotypes among patients with CRC.

Genotype	Stage I, n (%)	Stage II, n (%)	Stage III, n (%)	Stage IV, n (%)	P-value
A/A	29 (64.4)	38 (67.9)	49 (75.4)	41 (75.9)	0.483
A/G	16 (35.6)	18 (32.1)	16 (24.6)	13 (24.1)	-

EGF, epidermal growth factor; CRC, colorectal cancer.
